# Dosimetric Comparison, Treatment Efficiency Estimation, and Biological Evaluation of Popular Stereotactic Radiosurgery Options in Treating Single Small Brain Metastasis

**DOI:** 10.3389/fonc.2021.716152

**Published:** 2021-08-05

**Authors:** Yanhua Duan, Hongbin Cao, Boheng Wu, Yinghui Wu, Dong Liu, Lijun Zhou, Aihui Feng, Hao Wang, Hua Chen, Hengle Gu, Yan Shao, Ying Huang, Yang Lin, Kui Ma, Xiaolong Fu, Hong Fu, Qing Kong, Zhiyong Xu

**Affiliations:** ^1^Department of Radiation Oncology, Shanghai Chest Hospital, Shanghai Jiao Tong University, Shanghai, China; ^2^Department of Radiation Oncology, Renji Hospital, Shanghai Jiao Tong University, Shanghai, China; ^3^Nuclear Protective Treatment Department of Radiation, Navy Specialty Medical Center, Shanghai, China; ^4^Varian Medical Systems, Inc., Beijing, China; ^5^Department of Radiation Oncology, Fudan University Shanghai Cancer Center, Shanghai, China; ^6^Department of Mathematics and Information Technology, The Education University of Hong Kong, Hong Kong, China; ^7^Institute of Modern Physics, Fudan University, Shanghai, China

**Keywords:** biological evaluation, SRS, brain metastasis, Gamma Knife, cone, CRT

## Abstract

**Objectives:**

This study aimed to show the advantages of each stereotactic radiosurgery (SRS) treatment option for single small brain metastasis among Gamma Knife (GK), Cone-based VMAT (Cone-VMAT), and MLC-based CRT (MLC-CRT) plans.

**Materials and Methods:**

GK, Cone-VMAT, and MLC-CRT SRS plans were retrospectively generated for 11 patients with single small brain metastasis whose volume of gross tumor volume (GTV) ranged from 0.18 to 0.76 cc (median volume 0.60 cc). Dosimetric parameters, treatment efficiency, and biological parameters of the three techniques were compared and evaluated. The metric variation with the planning target volume (PTV) was also studied.

**Results:**

The conformity index (CI) was similar in GK and MLC-CRT plans, higher than Cone-VMAT. Cone-VMAT achieved comparable volume covered by 12 Gy (V12) and gradient index (GI) as GK, lower than MLC-CRT. The heterogeneity index (HI) of GK, Cone-VMAT, and MLC-CRT decreased sequentially. GK gave the lowest volume covered by 3 Gy (V3) and 6 Gy (V6), while MLC-CRT got the highest. The beam-on time and treatment time of GK, Cone-VMAT, and MLC-CRT decreased in turn. Tumor control probability (TCP) of all three SRS plans was greater than 98%, and normal tissue complication probability (NTCP) of all organs at risk (OARs) was below 0.01%. GK and Cone-VMAT resulted in superior TCP and NTCP of the normal brain tissue than MLC-CRT. The relative value of Cone-VMAT and GK for all metrics hardly changed with the target volume. Except for the unchanged HI and TCP, the other results of MLC-CRT with respect to GK improved as the target volume increased. MLC-CRT could produce higher CI than GK and Cone-VMAT when the target volume increased above 2 and 1.44 cc, respectively.

**Conclusion:**

For single small brain metastases, Cone-VMAT may be used as an alternative to GK-free centers. In addition to the advantage of short treatment time, MLC-CRT showed superiority in CI as the target volume increased. Treatment centers can choose appropriate SRS technique on a case-by-case basis according to institutional conditions and patients’ individual needs.

## Introduction

Brain metastasis is one of the most common brain tumors, and its morbidity and mortality are very high (1). Surgery and whole-brain radiation therapy (WBRT) are the traditional treatments of brain metastases (2). The development of radiotherapy enables stereotactic radiosurgery (SRS) to deliver high doses to brain metastases through a single fraction, giving the tumor ablative dose while minimizing organs at risk (OARs) damage. Thus, SRS can achieve a similar curative effect as surgery (3, 4). The introduction of Gamma Knife (GK) has made SRS a common treatment for small brain tumors (diameter <2–3 cm) (5–7). With the rapid development of radiotherapy equipment, the techniques based on linac have also achieved the therapeutic purposes of GK-SRS. The common solution of linac-based SRS is to use multiple beams to cover the target located at the isocenter (8) and delivered with either cone-based or multileaf collimator (MLC)-controlled ways.

There have been many studies comparing SRS/stereotactic radiotherapy (SRT) strategies for brain tumors (1, 9–16). These studies have compared the advantages and disadvantages of different SRS/SRT planning platforms for brain tumors. They have obtained many conclusions beneficial to clinical practice, which provide the basis for choosing radiotherapy techniques for brain tumors. However, the quality comparison of different SRS plans for single small brain tumors needs to be evaluated, especially the comparison among GK, cone-based volumetric-modulated arc therapy (VMAT), and MLC-based three-dimensional conformal radiotherapy (CRT). Although GK is a popular treatment technique for single small brain metastases (6, 7), not all hospitals are equipped with GK, and most hospitals only have linacs. Therefore, comparison results of GK and linac-based plans can provide necessary theoretical support for doctors to recommend treatment to patients. In addition, there are few studies on the biological differences in patients with single small brain metastasis using different SRS techniques. The biological effect caused by dosimetry is the patient’s final treatment result and the most concerning issue in clinical practice. Therefore, conducting biological research is critical and meaningful work.

This study evaluated the planning quality and biological effects of GK, cone-based VMAT (Cone-VMAT), and MLC-based CRT (MCL-CRT) delivered by the linac for a single brain metastasis. The relationship between the evaluation metrics and target volume was also studied. The results showed the advantages of each technology and provided data for selecting radiotherapy methods for patients with small single brain metastasis.

## Materials and Methods

### Patient Collection

This study retrospectively collected 20 patients with single brain metastases who received radiotherapy at Shanghai Chest Hospital from May 2019 to May 2020. Due to the hardware limitation of maximum aperture size, cone-based treatment is recommended for patients with tumors less than 1.5 cm. Therefore, nine patients with larger tumors were excluded. Finally, 11 cases were selected, including nine males and two females. The age of the patients ranged from 48 to 79 (median age 67 years) years old. The target size (maximum diameter) ranged from 0.95 to 1.43 cm (median size 1.30 cm), the GTV volume ranged from 0.18 to 0.76 cc (median volume 0.60 cc), and the clinical stage was T_1_N_0_M_0_. The planning target volume (PTV) volume ranged from 0.92 to 2.24 cc (median volume 1.85 cc). **Table 1** lists the details of all enrolled patients.

**Table 1:** Characteristics of enrolled cases.

Case	Gender	Age	Histological types	Prescription	Target size (cm)	Target Volume (cm^3^)	PTV volume (cm^3^)
1	Male	63	Adenocarcinoma	24Gy/1F	1.39	0.6	1.85
2	Male	69	Small-cell lung cancer	24Gy/1F	1.3	0.63	2.05
3	Male	57	Small-cell lung cancer	24Gy/1F	1.35	0.68	2.11
4	Male	63	Small-cell lung cancer	24Gy/1F	1.04	0.36	1.48
5	Male	79	Adenocarcinoma	24Gy/1F	1.3	0.65	2.03
6	Female	50	Adenocarcinoma	24Gy/1F	1.43	0.76	2.24
7	Female	72	Adenocarcinoma	24Gy/1F	1.39	0.65	2.01
8	Male	71	Adenocarcinoma	24Gy/1F	1.05	0.33	1.32
9	Male	67	Small-cell lung cancer	24Gy/1F	1.17	0.47	1.65
10	Male	76	Adenocarcinoma	24Gy/1F	0.95	0.18	0.92
11	Male	48	Small-cell lung cancer	24Gy/1F	0.97	0.28	1.25

Treatment targets and OARs were defined by experienced radiation oncologists from Shanghai Chest Hospital on the fusion images of high-resolution magnetic resonance imaging (MRI) and computed tomography (CT) with a slice thickness of 1 mm. Planning target volumes (PTV) were obtained by expanding 0.2 cm of GTV in three dimensions to take into account the uncertainty or movement during positioning and treatment (13). All structures were reviewed and approved by at least one independent experienced radiation oncologist and a neurosurgeon before being used for treatment planning design. When the study began, all selected patients signed informed consents and completed radiotherapy. This study was approved by the native Ethics Committee (the committee’s reference Number: KS1863).

### Radiation Dose

The recommendations of radiation oncology working group (RTOG) 90-05 (17) suggest that the SRS dose of brain metastases should be determined according to the size of lesions, varied from 15 to 24 Gy, and the prescribed dose should be 24 Gy for tumors <2 cm in maximum diameter. Therefore, the target dose of all cases in this study was 24 Gy delivered through one fraction. The constraints of OARs included optic nerve and chiasm maximum dose of 10 Gy and brainstem maximum dose of 12 Gy.

### Treatment Planning

A total of four institutions participated in the planning process. All patient images and contours were transferred to each planning workstation for retrospective planning design as follows. Three plans were created for each patient: (1) GK, (2) Cone-VMAT, and (3) MLC-CRT. Each plan was designed by an experienced physicist who was blind to the planning processes of the other modalities.

GK plans were designed and reviewed by two physicists with more than 5 years of experience from Renji Hospital and Navy Specialty Medical Center using the MASEP SuperPlan system (V4.2, MASEP instruments, Inc., Shenzhen, China) for SRRS head treatment equipment (MASEP instruments, Inc., Shenzhen, China). SRRS head treatment equipment has 25 Co-60 sources placed on four sectors. Each position corresponds to a different size collimator. Five different sizes of open collimators are available for every source in each sector (4, 8, 14, 18, and 22 mm), as well as a blocked collimator to conform to lesions of different shapes and sizes. Because each of the four sectors can move independently, it is possible to create plans with multiple composite shots where each sector is of a different collimator size. The GK plan started from manual shots filled in with composite small- to medium-sized collimators depending on the target volumes, followed by optimization. After the initial optimization, it usually achieves about 95% coverage with dose distribution that is not very conformal. Planner adjustments were then introduced to achieve 99% PTV coverage by the prescription dose and more conformal dose distribution. Adjustment included changing the position and weight of each existing shot and adding new shots.

The Cone-VMAT plans were done by a physicist from Shanghai Chest Hospital in collaboration with Varian’s Cone planning experts based on Eclipse Cone planning System (V13.5, Varian Medical Systems, Palo Alto, CA, USA) for an Edge™ linear accelerator (Varian Medical Systems, Palo Alto, CA, USA). The optional cone attachment sizes are 4, 5, 7.5, 10, 12.5, 15, and 17.5 mm in diameter, and the appropriate cone size was selected according to the tumor size. Considering the comprehensive factors such as the conformity of the target, dose gradient, and plan implementation, the same cone size was preferred for all fields of a plan. The mixed cone sizes would also be considered when the plan quality was poor. Cone-VMAT plans used five to nine non-coplanar arcs, and arc lengths and couch angles were set according to tumor location for an individual case. The Cone Dose Calculation (CDC) algorithm was used in Eclipse Cone Planning to calculate the dose for stereotactic cone applicators used in stereotactic radiosurgery (SRS) treatments with a dose calculated grid of 1.0 mm.

MLC-CRT plans were planned and reviewed by senior physicists from Shanghai Chest Hospital and Fudan University Shanghai Cancer Center using the Pinnacle Treatment Planning System (V9.10, Philips Radiation Oncology Systems, Fitchburg, WI, USA) for an Edge™ linear accelerator (Varian Medical Systems, Palo Alto, CA, USA) equipped with a high-definition HD 120 multileaf collimator (MLC)™. HD120 MLC™ has 120 leaves with a leaf width projected at the isocenter of 2.5 mm for the central 8.0 cm region and 5.0 mm for the two 7.0 cm peripheral regions (18, 19). The planning method was similar to our previous study (20). All CRT plans employed 10 or more 6MV fields, and the angular intervals of the fields were either 15 or 20 degrees. Collimator and couch angles were adjusted according to the individual situation. The collapsed cone convolution (CCC) algorithm was used for dose calculation with a calculation resolution of 1.0 mm.

### Dosimetric Comparison Metrics

In order to compare the results of different systems, the three-dimensional (3D) radiation doses of GK, Cone-VMAT, and MLC-CRT plans were exported in DICOM RT format to a third-party system, MIM Maestro Station (MIM Vista Corp, Cleveland, OH, USA). Here, the PTV coverage by the prescription dose was uniformly normalized to 99% across all of the platforms.

The dosimetric evaluation metrics used to compare different techniques are as follows: the dosimetric parameters of PTV included conformity index (CI), gradient index (GI), and heterogeneity index (HI).

CI (21) was computed as

(1) $$\text{CI}={{\text{V}}_{\text{T},\text{Rx}}}^{2}/{\left({\text{V}}_{\text{T}}*{\text{V}}_{\text{Rx}}\right)}^{2}$$

where V_T,Rx_ is the PTV volume covered by prescription dose, V_T_ is the target volume, and V_Rx_ is the volume covered by prescription dose. CI ranges from 0 to 1, and CI=1 indicates the best conformability.

GI (22) is calculated as

(2) $$\text{GI}={\text{V}}_{50\%\text{Rx}}/{\text{V}}_{\text{Rx}}$$

where V_50%Rx_ is the volume receiving half the prescription dose. A lower GI represents a faster dose falloff in normal tissue from the target.

HI (23) was defined as

(3) $$\text{HI}=({\text{D}}_{2}-{\text{D}}_{98})/{\text{D}}_{\text{Rx}}$$

where D_2_ and D_98_ correspond to doses delivered to 2 and 98% of the PTV volume, respectively. D_Rx_ is the prescription dose. Lower HI means a more uniform radiation distribution. The dosimetric parameters of OARs included the total dose volume to the normal brain tissues for 3 Gy (V3), 6 Gy (V6), and 12 Gy (V12).

### Treatment Efficiency Estimation

Beam-on time and estimated total treatment time were used to measure the treatment efficiency of the three platforms. Total treatment times for Cone-VMAT and MLC-CRT were estimated using the sum of patient setup time, image guidance and verification (IGRT) time, and radiation delivery time. Radiation delivery is based on the total dose divided by 1,400 cGy/min dose rate. Total treatment time for GK was estimated using the sum of patient setup time, shot transition time, and net beam-on time. Assuming the dose rate of new Co-60 sources is 360 cGy/min, the beam-on time was calculated by dividing the total dose by the dose rate. According to the treatment experience of centers relevant to this study on small brain lesions, the average setup time and image guidance time of previous patients were adopted.

### Biological Evaluation Metrics

To evaluate the clinical effects of different techniques, quantitative biological indices including TCP of the target and NTCP of OARs (optic nerves, optic chiasm, brainstem, and normal brain) were calculated using the Matlab program. The TCP was calculated based on the EUD model (24), and the details were as follows:

(4) $$EUD={\left[v_{i}D_{EQDi}\alpha^{\;}\right]}^{\frac{1}{\alpha }}$$

where *v_i_* is unitless and represents the *i*th partial volume receiving a dose *Di* in Gy, and *α* is the tumor normal tissue-specific parameter that describes the dose-volume effect. In this equation, *D_EQDi_* is the biologically equivalent physical dose of 2 Gy, defined as:

(5) $$D_{EQDi}=D_{i}\left(\frac{\alpha /\beta +d}{\alpha /\beta +2}\right)$$

where *d* is the dose per fraction of the treatment course. *α/β* is the tissue-specific linear-quadratic (LQ) parameter for the exposed organ. For brain lesions, tumor cells often have an α/β ratio of 10 Gy, whereas the α/β ratio for normal tissues is usually 3 Gy (25, 26).

Based on the EUD, the TCP can be calculated by

(6) $$TCP=\frac{1}{1+{\left(\frac{TCD_{50}}{EUD}\right)}^{4\gamma_{50}}}$$

where *TCD_50_*is the tumor dose to control 50% of the tumor when the tumor is homogeneously irradiated, and *γ_50_* is the change in TCP expected because of a 1% change in dose about the TCD_50_. The tumor-specific parameters *TCD_50_*, *γ_50_*, and *α* were cited from the study of Okunieff et al. (27), and the values were 51.77 Gy, 2.28, and −13, respectively.

NTCP was computed based on Lyman-Kutcher-Burman (LKB) model (28–30), and the details were as follows:

(7) $$NTCP=\frac{1}{\sqrt{2\pi }}\int_{-\infty }^{t}e^{-\frac{t^{2}}{2}dt}$$

(8) $$t=\frac{D_{eff}-TD_{50}}{mTD_{50}}$$

(9) $$D_{eff}={\left(\sum_{i}v_{i}D_{EQDi}{1/}^{n}\right)}^{n}$$

where *D_eff_* is the dose that, if given uniformly to the entire volume, will lead to the same NTCP as the actual non-uniform dose distribution (*D_eff_* is sometimes referred to as equivalent uniform dose, EUD), *TD_50_* is the uniform dose given to the entire organ volume that results in 50% complication risk, *m* is a measure of the slope of the sigmoid curve represented by the integral of the normal distribution, *n* is a parameter that describes the magnitude of the volume effect, and (*Di, vi*) are the bins of a differential dose-volume histogram (30). The calculation method of *D_EQDi_* was similar to formula (5). These parameters, *TD_50_*, *n*, and *m*, were obtained from listed data by Burman et al. (31) and are listed in **Table 2**.

**Table 2:** Normal tissue tolerance parameters for calculation of NTCP.

OARs	*TD_50_*	*n*	*m*
Optic nerves	65	0.25	0.14
Optic chiasm	65	0.25	0.14
Brainstem	65	0.16	0.14
Normal brain	60	0.25	0.15

### Statistical Analysis

Statistical analysis was performed using SPSS 22.0 (SPSS Inc., Armonk, NY, USA). The paired non-parametric Wilcoxon signed-rank test was used for comparisons between any two plans. A p less than 0.05 was considered statistically significant.

## Results

All SRS plans achieved clinically acceptable PTV coverage and OAR sparing.

### Case Example

**Figure 1:**
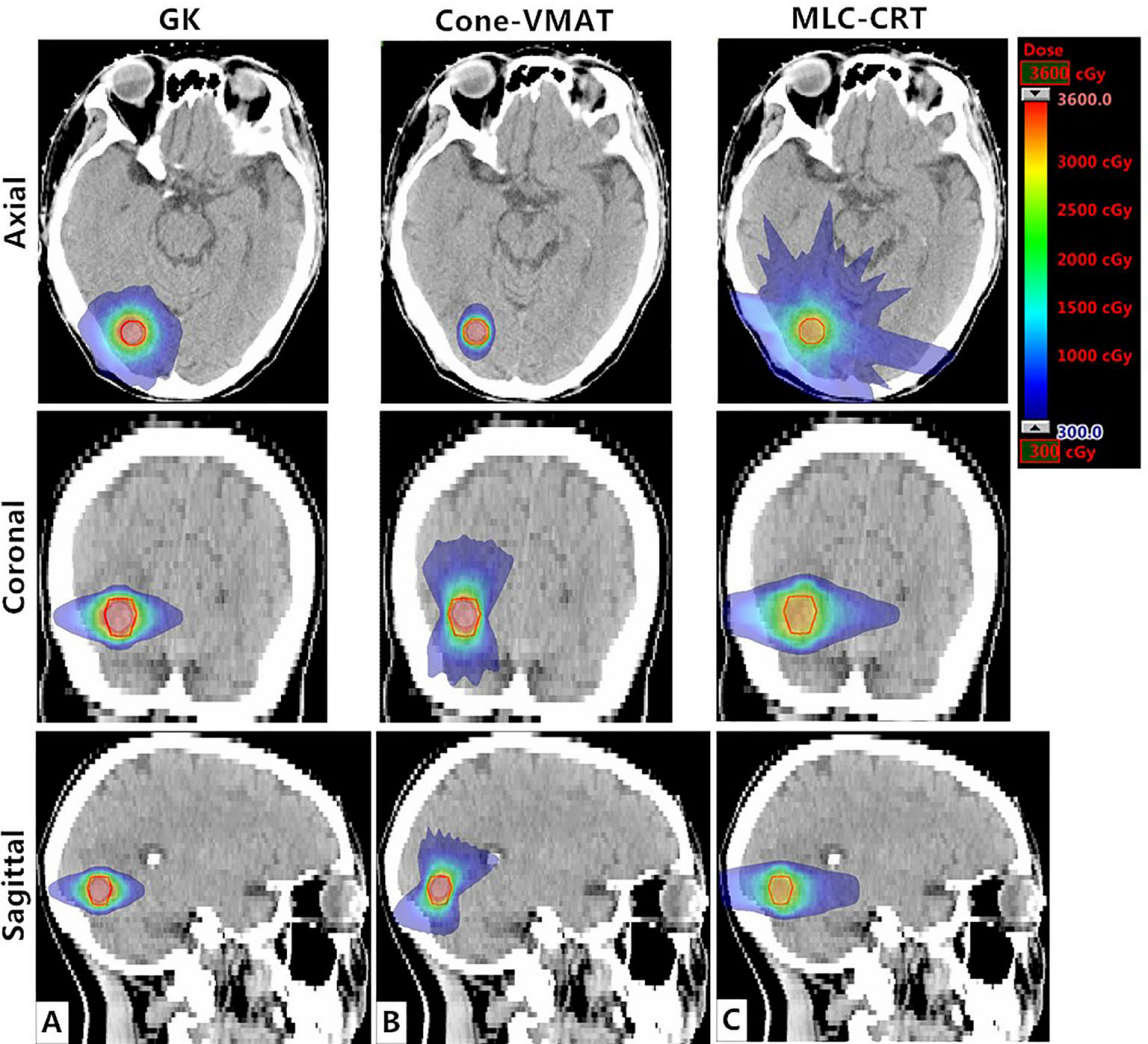
Axial, coronal, and sagittal cuts at the target center of the example case to visually demonstrate differences in dose distribution among **(A)** GK, **(B)** Cone-VMAT, and **(C)** MLC-CRT SRS plans. The solid red line represents PTV.

**Figure 1** demonstrates the dose distribution of a single case example across three different treatment modalities. Combined with axial, coronal, and sagittal cuts, V3 and V6 generated by GK were the smallest, while these of MLC-CRT were the largest. GK and Cone-VMAT obtained similar V12, lower than MLC-CRT. The detailed dosimetric comparison is presented in **Table 3**.

**Table 3:** Comparison of dosimetric parameters from different plans.

Metrics	GK	Cone-VMAT	MLC-CRT	p		
				GK *vs* Cone-VMAT	Cone-VMAT *vs* MLC-CRT	GK *vs* MLC-CRT
CI	0.72 ± 0.04	0.62 ± 0.06	0.68 ± 0.09	0.006	0.049	0.115
GI	2.67 ± 0.07	2.66 ± 0.16	5.47 ± 1.08	0.328	0.003	0.003
HI	1.08 ± 0.07	0.49 ± 0.06	0.29 ± 0.05	0.003	0.004	0.003
V3 (cc)	28.80 ± 10.19	34.92 ± 9.31	108.64 ± 27.31	0.008	0.003	0.003
V6 (cc)	10.26 ± 3.56	12.27 ± 3.72	41.64 ± 8.78	0.016	0.003	0.016
V12 (cc)	3.37 ± 1.24	3.45 ± 1.12	10.97 ± 1.26	0.657	0.003	0.003

### Dosimetric Comparison

**Table 3** shows the detailed dosimetric comparison on target as GI, CI, and HI. Among the three treatment methods, the CIs obtained by GK and MLC-CRT were similar (p = 0.115), which were 0.72 ± 0.04 and 0.68 ± 0.09, respectively, better than that of Cone-VMAT (0.62 ± 0.06) (p < 0.05). The GIs were comparable using GK and Cone-VMAT with 2.67 ± 0.07 and 2.66 ± 0.16 (p = 0.328), significantly lower than MLC-CRT (5.47 ± 1.08) (p < 0.05). The HI from GK was the highest (1.08 ± 0.07), followed by Cone-VMAT (0.49 ± 0.06) and the lowest with MLC-CRT (0.29 ± 0.05), and statistical differences were found between any two plans (p < 0.05).

Doses to the normal brain as V3, V6, and V12 are also listed in **Table 3**. GK produces the smallest V3 and V6, followed by Cone-VMAT, and MLC-CRT results in the largest V3 and V6. There are statistical differences between the three plans (p <0.05). For intermediate-dose V12, GK and Cone-VMAT showed no difference (p = 0.657), which were 3.37 ± 1.24 and 3.45 ± 1.12, respectively, lower than MLC-CRT (10.97 ± 1.26) (p = 0.003).

### Treatment Efficiency Estimation

The beam-on time and estimated total treatment time for the three plans are listed in **Table 4**. The beam-on time of GK was 26.67 ± 5.35 min, significantly longer than that of Cone-VMAT and MLC-CRT (p = 0.003). The total treatment time of GK, Cone-VMAT, and MLC-CRT decreased sequentially (p = 0.003), which were 38.64 ± 5.51, 28.14 ± 0.93, and 18.56 ± 0.47 min, respectively.

**Table 4:** Treatment efficiency estimation of different plans.

Metrics (min)	GK	Cone-VMAT	MLC-CRT	p		
				GK *vs* Cone-VMAT	Cone-VMAT *vs* MLC-CRT	GK *vs* MLC-CRT
Beam-on time	26.67 ± 5.35	3.88 ± 0.39	3.14 ± 0.27	0.003	0.003	0.003
Total treatment time	38.64 ± 5.51	28.14 ± 0.93	18.56 ± 0.47	0.003	0.003	0.003

### Biological Evaluation

**Table 5** shows the TCP of target and NTCP of OARs. All the three planning methods obtained high and close mean TCP. TCP of GK (99.76 ± 0.11) was similar to that of Cone-VMAT (99.61 ± 0.08) (p = 0.051), significantly higher than that of MLC-CRT (98.41 ± 0.32) (p = 0.003). NTCPs of all OARs were less than 0.01%. There was no statistical difference between any two plans for NTCPs of the optic nerve, optic chiasm, and brainstem (p > 0.05). GK and Cone-VMAT produced comparable NTCP of normal brain tissues (p = 0.131), where the mean values were 1.14 × 10^-8^% and 1.53 × 10^-8^% respectively, significantly lower than that of MLC-CRT (p = 0.004 and 0.003), whose result was 5.84 × 10^-8^%.

**Table 5:** Comparison of TCP and NTCP from different plans.

Metrics (%)		GK	Cone-VMAT	MLC-CRT	p		
					GK *vs* Cone-VMAT	Cone-VMAT *vs* MLC-CRT	GK *vs* MLC-CRT
TCP		99.76 ± 0.11	99.61 ± 0.08	98.41 ± 0.32	0.051	0.003	0.003
NTCP	Optic nerves	<0.01	<0.01	<0.01	0.530	0.330	0.248
	Optic chiasm	<0.01	<0.01	<0.01	0.345	0.091	0.056
	Brainstem	<0.01	<0.01	<0.01	0.477	0.657	0.213
	Normal brain tissue	<0.01	<0.01	<0.01	0.131	0.004	0.003

### Variation Dependence on the Target Volume

**Figure 2:**
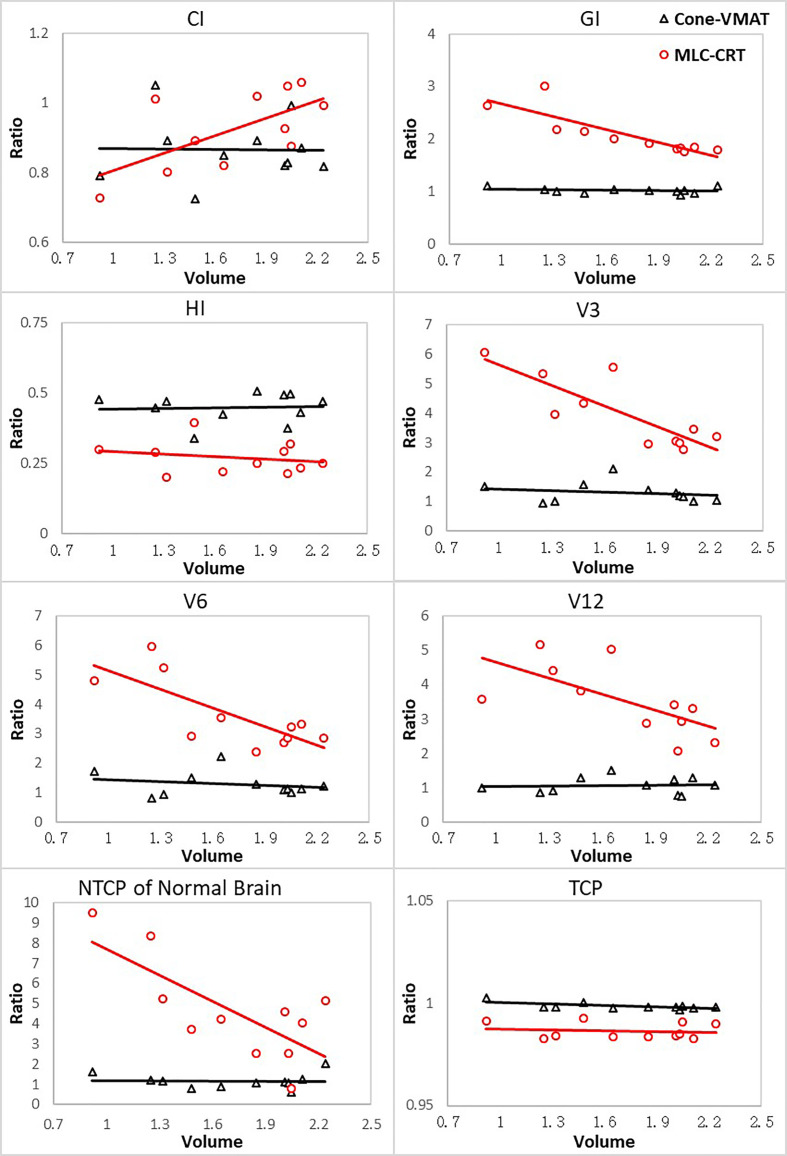
The ratios of parameters for other two techniques relative to GK with respect to different PTV volumes. Volumes are in cm^3^.

This study also explored the relationship between the PTV volumes and the evaluation metrics with statistical differences, including CI, GI, HI, V3, V6, V12, TCP, and NTCP of the normal brain. The variations of the other two modalities relative to the GK (measured as the ratio to that of GK) with different PTV volumes are plotted in **Figure 2**.

For all evaluation parameters, the relative values of Cone-VMAT to GK hardly changed with the PTV volume. For GI, V3, V6, V12, NTCP of the normal brain tissue, GK and Cone-VMAT performed better than MLC-CRT, but these differences decreased with the PTV volume increasing. For CI, MLC-CRT showed better conformability than GK and Cone-VMAT when PTV volume was greater than 2 and 1.4 cc, respectively. For HI and TCP, the relative performance of the three techniques was almost independent of the PTV volume.

## Discussion

This study compared the SRS’s plan quality and biological effect in treating single small brain metastasis across three platforms: GK, linac-based Cone-VMAT, and MLC-CRT. The overall findings of this study have demonstrated that the three commercially available SRS options can achieve clinically acceptable plans. The results of this study have revealed much interesting information. Compared with the other two plans, MLC-CRT had almost no dosimetric and biological advantages but with the shortest treatment time. GK had some advantages in dosimetry and biology, while that was at the cost of the longest treatment time. Unexpectedly, MLC-CRT resulted in a similar CI to GK for single small brain metastasis, and Cone-VMAT could compete with GK in terms of V12, GI, TCP, and NTCP of the brain. For the metrics with statistical differences, the relative performance of Cone-VMAT and GK was unrelated to the PTV volume. The performance of MLC-CRT improved with the increase of PTV volume (except for HI and TCP). The relative advantages of the three techniques for CI depended on the PTV volume. Overall, to the best of our knowledge, this is the first report quantifying the quality and biological differences among GK, Cone-VMAT, and MCL-CRT to treat single small brain metastasis. Despite these statistical differences, the clinical relevance and impact of such differences remain to be determined.

GK and MLC-CRT obtained similar CI, which was higher than Cone-VMAT. The high conformity of GK and MLC-CRT could be explained by the mechanical design. GK could conform to the target by spherical focusing with far more non-coplanar fields than the other two techniques. MLC-CRT used HD120 MLC with high precision for dose painting. However, Cone-VMAT delivered by Edge accelerator was generated with multiple arc beams controlled by cones of different sizes. As a result, the three-dimensional dose distribution was generally spherical or ellipsoidal, which greatly limited the conformity of targets with an irregular shape, resulting in low CI.

A risk assessment study about the low-dose area of normal brain tissue in brain tumor patients treated with SRS showed no increased risk of malignancy compared to the general population within 5 years (32). While V12 has been reported as one of predictors for radiation induced necrosis after intracranial single-fraction SRS (33–35). Although GK performed best in V3 and V6, showing its potential ability of dose falloff, V12 seems to be a more critical parameter proven by existing studies. It was certainly unexpected to see that Cone-VMAT could obtain a similar V12 to GK, which resulted in the comparable GI with GK (GI = V_50%Rx_/V_Rx_ = V_12_/V_Rx_), better than MLC-CRT. We have not yet found similar studies for single small metastasis, but some of our findings are consistent with previous studies (1, 13, 36). These studies showed that GK and MLC-based plans resulted in similar V12 or GI in multiple brain metastases or large intracranial tumors. This sharp falloff of GK benefited from the physical design, which allowed for thousands of non-coplanar beams focusing on a single target. Cone-VMAT consisted of multiple arc beams, while MLC-CRT had the least complicated beam sets. The advantage of the multibeam angle is evident, as the GI is lower for GK and Cone-VAMT, followed by MLC-CRT. Besides beam number, the difference in GI of the three methods was also related to the physical machine design. The nominal source-to-axis distance (SAD) is 50 cm for GK, compared to 100 cm for linac. Due to the cone attachment, the Cone-VMAT has a lower source-to-skin distance (SSD) than MLC-CRT. Therefore, the MLC-delivered plan has a flatter lateral physical penumbra, resulting in a greater GI. Note that for single-fraction cases with target close to OARs, we recommend the GK or Cone-VMAT treatments with a sharper dose gradient to achieve the maximum OAR sparing. For fractionated cases, considering the uncertainty of location, the final choice should be made after careful consideration of more other metrics.

The sequentially decreased HI of GK, Cone-VMAT, and MLC-CRT indicates a decrease in their hotspots. Some studies got similar conclusions. Nakazawa’s study (37) showed that the mean HI was significantly larger for GK than for linac-MLC skull base tumor plans. Despite minimal supporting clinical data, theoretically, with the same peripheral dose, a higher dose inside the tumor may translate to enhanced clinical efficacy in treating hypoxic tumors (13). Due to negligible intrafraction motion with frame-based treatment, high HI could even be considered an advantage of traditional single-fraction GK treatment. However, dose homogeneity may be critical for fractionated cases as intra- and inter-fraction uncertainties or shifts could cause serious adverse effects when hotspots occur near sensitive regions. HI needs to be focused on at that time, and MLC-CRT may provide a relatively safe solution.

Treatment efficiency should also be considered. We found that the beam-on time of GK was much longer than those of two linac-based plans, which indicates that some dosimetric advantages come at the cost of longer beam-on times. The delivery efficiency of GK can be improved by using a large helmet size and less complex plans with fewer shots. However, this will result in a lower conformity index and increase the low-dose spread (1). GK had the longest total treatment time; thus, additional tolerance assessment might be needed for patients with weak health or poor self-control ability. MLC-CRT had apparent advantages in terms of treatment time, which would reduce the possibility of involuntary patient movement during treatment. Moreover, for developing countries with large populations, some treatment institutions cannot meet the demand of a huge number of patients even though the machine runs day and night, and MLC-CRT with shorter treatment time is undoubtedly an effective solution for these centers. In addition, only few types of linacs are equipped with cone devices, and MLC-based SRS is usually the only choice for many centers without other treatment equipment except linacs. It should be noted that the estimated total treatment time was obtained according to the setup and verification time of the centers involved in this study. Due to the differences in many factors such as the number of patients, staffing, and medical conditions, the treatment time in different centers will be greatly different. However, the relative trend of the three SRS techniques is the same.

A novel and interesting result of this study was that Cone-VMAT and GK had similar TCP, which was higher than that of MLC-CRT. Nevertheless, the TCPs of the three techniques were all above 98%, indicating their good performance on tumor control. Although there were no results that can be directly compared with this study. Kumar T et al. found that GK achieved TCP ranging from 98.75 to 100% for single and multiple brain tumors (38). Pasciuti K et al. obtained an overall mean TCP of 0.95, ranging from 0.89 to 1 for brain metastases treated with CRT (39). Furthermore, the successively decreasing TCP of GK, Cone-VMAT, and MLC-CRT (see **Table 5**) came from the decreasing hotspots (see HI in **Table 3**) in the target. For the same type of tumor, the TCP caused by radiotherapy is affected by the actual radiation dose of the tumor, which is closely related to the prescription dose, coverage, and hotspots in the target. On the premise that the three techniques used the same prescription dose and were normalized to the same target coverage, the difference of TCP was mainly due to the different hotspots in the target.

The NTCPs of almost all OARs that resulted in this study were less than 10^-10^. Thus, all three techniques could achieve good complication control effects. Similar to our results, the NTCP obtained by Kumar T et al. (38) was 10^-10^ to 10^-8^ from GK plans for single and multiple brain tumors. Pasciuti K et al. (39) found that when the NTCP was calculated for brain and brainstem close to lesions, the mean NTCP was 5.55 × 10^-3^ with a median of 1.26 × 10^-5^. We did not find a statistical difference in NTCP of optic nerves, optic chiasm, and brainstem (see **Table 5**), which was caused by the great difference in the field settings of the three techniques. Many factors, such as dosimetry, delivery efficiency, and hardware requirements for actual implementation, need to be considered in the planning process. GK needs to reduce the number of shots as much as possible on the premise of adequate target irradiation to reduce the treatment time. Cone-VMAT and MLC-CR should optimize CI, GI, etc., while avoiding the collisions of gantry and couch. The above restrictions make the field settings of the three techniques intricate. For the same patient, it is hard to determine the relative positional relationship between the field of each technique and OAR. Therefore, which technique would cause the maximum dose of an OAR was unknown, resulting in no difference in the NTCP of optic nerves, optic chiasm, and brainstem. However, we found that GK and Cone-VMAT got comparable NTCP of the normal brain tissue, which was slightly lower than MLC-CRT, but the numerical difference was less than 5 × 10^-10^. The possible reason was that the faster dose falloff resulted in lower NTCP of normal brain (see GI in **Table 3** and NTCP in **Table 5** for details).

In the results of parameters variation with the PTV volume, the relative values of Cone-VMAT and GK hardly changed as the target volume increased for all criteria. The main reason may be that Cone-VMAT performed dose-painting based on cone accessories with different sizes, similar to GK. Compared with the other two techniques, the performance of MLC-CRT in GI, V3, V6, V12, NTCP of normal brain and CI gradually improved with the increasing target volume, and the CI of MLC-CRT was better than GK and Cone-VMAT when PTV volume increased above 2 and 1.4 cc, respectively. The possible reason was that the width of the HD120 MLC™ at the isocenter used for CRT plan was 2.5 mm, which determined that the larger the tumor volume, the better CRT’s performance on dose falloff and conformity. Although the tumor types and fractionation schemes were different from this study, a study for brain metastases and gliomas also showed that the MLC-based plan produced better dose distribution and lower integral dose to the brain than GK for the target with a larger volume. Vergalasova I et al. (40) also found the improved conformity of the MLC-based SRS techniques over GK for large PTV volumes of multiple brain metastases. For HI and TCP, no relative changes between any two techniques existed with various target volumes. Because HI (hotspots) was the main influencing factor of TCP in this study, the relative trend above was consistent with the theoretical expectation.

Despite the high precision of these SRS instruments, a PTV margin is still required before the treatment planning process. A typical PTV margin includes a combination of targeting uncertainty, image system uncertainty, intra-/inter-fractionation motion, and setup uncertainties. Traditionally, single-fraction GK treatment needs no PTV margin due to submillimeter targeting accuracy and minimal frameshift (13). Cone-based SRS uses a PTV margin of 0–2 mm typically. For MLC-based SRS, an empirical isotropic PTV margin of 2 mm is usually used (13). For direct comparison, a 2 mm PTV margin was applied for all modalities in this study. Note, the PTV margin increased the target volume, which may increase the risk of neurological morbidity from radiation necrosis. Thus, users should carefully determine the optimal PTV margin for SRS treatment.

Another factor to consider is the planning goal of the target coverage. A minimal 99% coverage is required for GK planning, and minimal 95% coverage for regular photon treatment in our clinical practice. In this study, all plans were renormalized to 99% PTV coverage by changing the prescription isodose for direct comparison. For example, the original MLC-CRT plans prescribed with 82% isodose to ensure 95% coverage can be adjusted to 80% isodose for 99% target coverage. This adjustment will make the overall dose in the Cone-VMAT and MLC-CRT plans a few percentages higher while has little effect on GK. In clinical practice, where the target coverage could vary, the overall trend of the dosimetric parameters *versus* target volume across different platforms will remain the same.

Here are some limitations and prospects of this research. As a retrospective study, patients were not randomized, which resulted in a potential selection bias. Also, the effect of the radiation dose rate was not accounted for in this study, with different treatment methods having quite varied maximum delivery rates that may lead to different biological responses. Future studies can be carried out. It is necessary to consider whether the dosimetric and biological differences of currently available SRS techniques actually have clinically tangible impacts, which would require multi-institutional prospective clinical trials with long-term follow-up (40). Unfortunately, the mean survival time of patients with brain metastases is short, which makes follow-up studies quite difficult. In addition, adding biological factors into the optimization stage of treatment plans can allow doctors to understand the prognostic effect of patients intuitively. There have been some studies (41–45) involving the concept of biological optimization. However, research in this area is still in the initial stage, and it will take a long way to replace physical optimization with biological optimization. Besides, the research objectives in this study are patients with a single lesion. The conclusion may be different if cases with multi-lesions are studied. Further investigation is required to analyze the difference between the popular SRS techniques for patients with multiple metastatic lesions. Finally, this study aims to evaluate the planning quality and biological differences among GK, Cone-VMAT, and MLC-CRT. It provides a benchmark for understanding the superiority of one technology over another. Before choosing the most suitable SRS method, factors such as dosimetry, delivery efficiency, and clinical situation must be comprehensively evaluated.

## Conclusion

Overall, this study provides useful dosimetric, treatment efficiency, and biological insights of GK, Cone-VMAT, and MLC-CRT for single small brain metastasis. The results show that GK outperformed the other two techniques across V3 and V6, but all essentially at the cost of longer beam-on times. Cone-VMAT is comparable to GK in V12, GI, TCP, and NTCP of the normal brain tissue and may be used as a treatment alternative to GK for single small brain metastases. MLC-CRT is not outstanding in dosimetry and biology but has obvious advantages in shortening the treatment time. In addition, the relative performance of Cone-VMAT and GK is independent of the target volume. Most results of MLC-CRT with respect to GK improved with the increase of target volume except for the almost unchanged HI and TCP. MLC-CRT results in higher CI than GK and Cone-VMAT when the target volume increases over 2 and 1.44 cc, respectively. Treatment centers should choose appropriate SRS methods individually for patients with single small brain metastasis after comprehensive evaluation of dosimetry, delivery efficiency, and clinical factors.

## Data Availability Statement

The raw data supporting the conclusions of this article will be made available by the authors, without undue reservation.

## Ethics Statement

The studies involving human participants were reviewed and approved by Ethics Committee of Shanghai Chest Hospital (the committee’s reference Number: KS1863). The patients/participants provided their written informed consent to participate in this study. Written informed consent was obtained from the individual(s) for the publication of any potentially identifiable images or data included in this article.

## Author Contributions

YHD: Conceptualization, Methodology, Software, Formal analysis, Investigation, Resources, Data Curation, Writing – Original Draft, Visualization. HBC: Validation, Formal analysis, Writing – Review & Editing, Supervision, Project administration. YHW, BHW, LJZ: Methodology, Software, Validation, Data Curation, Writing – Review & Editing, Visualization. DL: Methodology, Validation, Software, Writing -Review & Editing. AHF: Software, Validation, Writing – Review & Editing. HW, HC, HLG, YS, YH, YL: Methodology, Validation, Writing – Review & Editing. KM: Validation, Software, Visualization, Writing -Review & Editing. XLF: Validation, Writing – Review & Editing, Project administration. HF, QK: Validation, Writing – Review & Editing, Supervision. ZYX: Validation, Writing – Review & Editing, Supervision, Funding acquisition. All authors contributed to the article and approved the submitted version.

## Funding

Nurture projects for basic research of Shanghai Chest Hospital (No. 2019YNJCM05).

## Conflict of Interest

DL and KM were employed by Varian Medical Systems.

The remaining authors declare that the research was conducted in the absence of any commercial or financial relationships that could be construed as a potential conflict of interest.

## Publisher’s Note

All claims expressed in this article are solely those of the authors and do not necessarily represent those of their affiliated organizations, or those of the publisher, the editors and the reviewers. Any product that may be evaluated in this article, or claim that may be made by its manufacturer, is not guaranteed or endorsed by the publisher.
